# Ethnic variations in referrals to the Leicester memory and dementia assessment service, 2010 to 2017

**DOI:** 10.1192/bjo.2020.69

**Published:** 2020-08-03

**Authors:** Andrew Wilson, John Bankart, Emma Regen, Kay Phelps, Shona Agarwal, Mark Johnson, Raghu Raghavan, Bina Sitaram, Hari Subramaniam

**Affiliations:** Department of Health Sciences, University of Leicester, UK; Department of Health Sciences, University of Leicester, UK; Department of Health Sciences, University of Leicester, UK; Department of Health Sciences, University of Leicester, UK; Department of Health Sciences, University of Leicester, UK; De Montfort University, UK; Department of Health Sciences, University of Leicester, UK; Leicestershire Partnership NHS Trust, UK

**Keywords:** Dementia, primary care, referral, BAME, diagnosis

## Abstract

**Background:**

The incidence of dementia in Black, Asian and minority ethnic (BAME) groups is increasing in the UK, with concern about underdiagnosis and late presentation.

**Aims:**

By reviewing referrals to memory clinics from Leicester City we examined whether the following differed by ethnicity: the proportion with a diagnosis of dementia, type of dementia and severity at presentation.

**Method:**

We examined referrals between 2010 and 2017: all those whose ethnicity was recorded as Black (*n* = 131) and a random sample of 260 Asian and 259 White British referrals. Severity of dementia was assessed by record review. Odds ratios (ORs) were adjusted for general practice, age, gender and year of referral.

**Results:**

A diagnosis of dementia was recorded in 193 (74.5%) White British, 96 (73.3%) Black and 160 (61.5%) Asian referrals. Compared with Asians, White British had twice the adjusted odds of a dementia diagnosis (OR = 1.99 (1.23–3.22). Of those with dementia, Alzheimer's disease was more common in White British (57.0%) than in Asian (43.8%) and Black referrals (51.0%): adjusted OR White British versus Asian 1.76 (1.11–2.77). Of those with dementia, the proportion with moderate/severe disease was highest in White British (66.8%), compared with 61.9% in Asian and 45.8% in Black groups. The adjusted OR for the White versus Black groups was 2.03 (1.10–3.72), with no significant difference between Asian and White British groups.

**Conclusions:**

Differences in confirmed dementia suggest general practitioners have a lower threshold for referral for possible dementia in some BAME groups. Unlike other centres, we found no evidence of greater severity at presentation in Asian and Black groups.

## The problem

There are more than 25 000 people with dementia from Black Asian and Minority Ethnic (BAME) groups in the UK.^1^ This number is expected to increase sevenfold to over 172 000 people by 2051, compared with a twofold increase in the number of people with dementia across the total population in the same time period.^2^ This represents a major challenge in providing culturally appropriate health and social care, especially in places such as Leicester with high BAME populations.

## Previous research

Internationally, there is evidence for underdiagnosis of dementia in BAME groups. A systematic review (drawing largely on US studies) concluded these groups accessed diagnostic services later (i.e. symptoms were more severe at presentation), and were less likely to be prescribed cholinesterase inhibitor drugs.^3^ In the UK incidence of dementia in ethnic groups has been examined using one of its largest general practice databases, which is broadly representative of the UK population. It was found that in Asians the incidence of diagnosis was lower than in the White population (adjusted incidence rate ratio [IRR] = 0.82 for women and IRR = 0.88 for men). Rates of diagnosis were higher in the Black than the White population (IRR = 1.25 for women, IRR = 1.28 for men). The authors concluded that it was unclear whether lower rates in the Asian population were because of lower incidence or underdiagnosis, but that the rate of diagnosis in the Black population, despite being higher, still suggested underdiagnosis because of the higher levels of risk factors for dementia, (including diabetes and obesity, as well as socioeconomic predictors) in this group.^4^ To our knowledge, only two UK studies have examined severity of dementia at presentation to memory clinics by ethnicity. In a large study of over 10 000 patients referred to two mental health trusts, Mukadam et al found Black and Asian patients had lower cognitive score at diagnosis,^5^ and a smaller study found lower scores at diagnosis in Black patients.^6^

There are several steps in the pathway to diagnosis of dementia. First, the patient has to seek help from a healthcare professional, usually their general practitioner (GP). Second, the healthcare professional has to assess cognition, and if dementia is suspected refer the patient for a specialist to confirm or refute the diagnosis. Both these steps may be more problematic for some people from BAME groups.^7–12^

## Local setting

Leicester is one of the most ethnically diverse cities in the UK. In the 2011 census, 50.5% of the population was White (45.1% White British), 37.1% Asian or Asian British (28.3% Indian, 2.5% Pakistani, 1.1% Bangladeshi) and 6.2% Black or Black British (3.8% African, 1.5% Caribbean).^13^

In this study we examined referrals from Leicester City Clinical Commissioning Group to a district wide memory service to establish whether the following differed by broad ethnic group: the proportion with a diagnosis of dementia confirmed, type of dementia, severity at presentation and use of cholinesterase inhibitor drugs when indicated. It was beyond the scope of this project to estimate referral rates by ethnicity as we did not have access to age-stratified population data by ethnicity. In a related study using 2011 census data we found that, in Leicester City, after adjusting for age, there were no differences between Asians and White British in their odds of referral to the memory clinic from 2011 to 2013, but from 2014 to 2017, members of Asian groups had higher odds of being referred.^14^

## Method

Leicestershire Partnership NHS Trust maintains a database of all referrals to its memory clinic. This was started in 2010 and fields include ethnicity (using standard National Health Service (NHS) codes based on UK census categories),^15^ other demographic details, clinical findings and diagnosis. Ethnicity was self-ascribed, either in the GP practice before referral or at the memory clinic if this information was not provided in the referral letter. The main categories of ethnicity are: White (subcategories British, Irish and other White), Mixed, Asian or Asian British, Black or Black British, and other ethnic groups (including Chinese).

In 6.4% of records ethnicity was recorded as ‘not known’ and in 9.6% as ‘not stated’. We extracted data collected on all 3602 patients whose ethnicity was recorded as White British, Asian/Asian British or Black/Black British from Leicester City Clinical Commissioning Group from January 2010 to December 2017. Other ethnic categories, including, for example, Mixed, Chinese and Irish were not examined.

We had planned to use the Mini-Mental State Examination (MMSE) score to document severity of dementia but in too many cases this was missing. Missing values were 41% in Asian groups and 39% in Black groups (not a statistically significant difference, *P* = 0.8). We therefore decided not to use this variable, so there was no need to assess missing values in White British. Therefore, to assess severity, two trained senior psychiatric nurses (one band 8 and another band 6) went through the clinical information in the case notes and electronic records together (including the MMSE score if present) and jointly agreed on a combined clinical dementia severity score (none, mild, moderate, severe).

The following definitions were used: mild, memory impairment/cognitive dysfunctions with mild functional impairment not needing additional support; moderate, as above but with evidence of functional impairment needing support; and severe, significant memory/cognitive impairment plus significant functional impairment or behavioural and psychological symptoms of dementia.

Disagreements in classification of severity were resolved through discussion. Data were extracted by members of the clinical team who had access to the full clinical records: the research team received only an anonymised data-set. The ethics committee agreed that in these circumstances, informed consent was not needed (see ethics statement below).

Analysing all records was beyond our financial and clinical resources. We included all 131 referrals with Black ethnicity and selected a random sample of 259/2525 patients with White British ethnicity and a random sample of 260/946 patients with Asian ethnicity. We originally sampled 260 White British but 1 patient was later excluded as they were not referred by a practice in Leicester. Using ordinal regression, this gave 80% power to detect an odds ratio of 1.7 in severity between Black and White British groups and Black and Asian groups, with greater power to detect differences between White and Asian groups.

Simple randomisation was used to derive each sample separately and was carried out using Stata v14.

### Ethics statement

The authors assert that all procedures contributing to this work comply with the ethical standards of 147 the relevant national and institutional committees on human experimentation and with the Helsinki 148 Declaration of 1975, as revised in 2008. All procedures involving patients were 149 approved by University of Leicester Ethics Sub-Committee for Medicine and Biological Sciences, 150 reference: 13597-sa144-healthsciences.

### Statistical methods

#### Descriptive statistics

We present mean age (s.d.) and age bands and gender (*n*, %), and proportions with dementia, type of dementia and other diagnoses by ethnicity.

#### Inferential statistics

Multivariable multilevel logistic regression models were used to adjust for age (continuous), gender and year, in order to generate adjusted odds ratios (ORs, with 95% CIs) to compare ethnic groups. Patients were clustered within general practices, so we adjusted for this by fitting multilevel models with random intercepts and slopes. Year was modelled as a linear function with a quadratic check for nonlinearity. *P* = 0.05 was set as the significance level for the overall effect of the ethnic variable but the *post hoc* comparisons were subjected to a Bonferroni correction for multiple comparisons, so *P* < 0.017 (*P* = 0.05/3) was the amended significance level. Analyses were carried out in Stata 14.

We assessed three null hypotheses:

- In those with a specialist diagnosis of dementia, there will be no difference between ethnic groups in the odds of being diagnosed with more severe dementia versus less severe dementia. Moderate and severe categories were combined owing to sparse data leading to some empty cells.^16^
- In those with any diagnosis, there will be no difference between ethnic groups in the odds of being diagnosed with dementia versus any other diagnosis
- In those with a dementia diagnosis, there will be no difference between ethnic groups in the odds of being diagnosed with Alzheimer's disease versus any other dementia

## Results

A total of 650 records from 2010 to 2017 were examined, comprising all available referrals of Black groups and random samples of White British and Asians. The total sample comprised 370 females (57%), 279 males (43%), with 1 missing value for gender. Mean age for the whole sample was 77.1 years (s.d. = 9.8). Age and gender by ethnicity are shown in Table 1. The proportion of patients aged ≥85 was much higher in White British than other groups. Composition of the sample by year was as follows: 2010 (*n* = 8, 1%), 2011 (*n* = 40, 6%), 2012 (*n* = 37, 6%) 2013 (*n* = 86, 14%) 2014 (*n* = 111, 18%) 2015 (*n* = 140, 22%) 2016 (*n* = 115, 18%) 2017 (*n* = 87, 14%). Patients were referred by 53 practices (reduced to 49 after covariate attrition), the number per practice ranging from 1 to 51.

**Table 1:** Demographics of the sample, severity of dementia, diagnosis and prescription of cholinesterase inhibitors by ethnicity

	White British groups	Asian groups (*n*, %)	Black groups (*n*, %)	*P*
Total sample, *n*	259	260	131	
Age, mean (s.d.)	79.8 (9.5)	74.7 (9.4)	76.4 (9.9)^a^	
Male, *n* (%)	70 (27.0)	64 (24.6)	45 (34.4)	
All dementia, *n* (%)	193 (74.5)	160 (61.5)	96 (73.3)	<0.001
Other diagnoses, *n* (%)	35 (13.5)	82 (31.5)	26 (19.8)	
Diagnosis missing, *n* (%)	31 (12.0)	18 (6.9)	9 (6.9)	
Age at diagnosis of dementia, mean (s.d.)	81.0 (8.4)	77.1 (8.8)	77.9 (9.1)^b^	
Age <75 years, *n* (%)	72 (27.8)	122 (46.9)	41 (31.3)	
Age 75–84, *n* (%)	102 (39.4)	102 (39.2)	66 (50.4)	
Age ≥85, *n* (%)	85 (32.8)	36 (13.8)	24 (18.3)	
Type of dementia, *n* (% all dementia)				
Alzheimer's disease	110 (57.0)	70 (43.8)	49 (51.0)	0.052
Vascular dementia	39 (20.2)	36 (22.5)	15 (15.6)	0.45
Mixed dementia	33 (17.1)	25 (15.6)	4 (4.2)	0.009
Other/unclassified dementia	11 (5.7)	29 (18.1)	28 (29.2)	<0.001
Severity of dementia, *n* (% all dementia)				
Mild	56 (29.0)	53 (33.1)	46 (47.9)	
Moderate	96 (49.7)	81 (50.6)	40 (41.7)	
Severe	33 (17.1)	18 (11.2)	4 (4.2)	
Severity unable to determined	8 (4.1)	8 (5.0)	6 (6.2)	
Other diagnoses, *n* (% total cases)				
Mild cognitive impairment	25 (9.7)	41 (15.8)	10 (7.6)	0.0026
Depression	4 (1.5)	14 (5.4)	4 (3.1)	0.052
Psychosis	1 (0.4)	9 (3.5)	8 (6.1)	0.009
Other	5 (1.9)	18 (6.9)	4 (3.1)	<0.001
Cholinesterase inhibitor drugs, *n* (% cases of Alzheimer's and mixed dementia)	123 (86.0)	83 (87.4)	43 (81.1)	0.57

a. Five missing.

b. Four missing.

Table 1 also shows diagnosis by ethnicity. Compared with the White British population, diagnoses other than dementia were more common in Asian and Black groups (13.5% (White British), 31.5% (Asian) and 19.8% (Black)). Among the non-dementia diagnoses, mild cognitive impairment and depression were highest in Asian groups, and psychosis in Black groups.

Table 1 also shows severity of dementia at ‘diagnosis’, which could be assessed in 427/449 (95.1%) records. In the remaining cases insufficient information was available to determine severity. The proportion with severe dementia was highest in the White British group: 17%, compared with 11% in Asian and 4% in Black groups. The proportion with mild dementia was correspondingly higher in Asian (33%) and Black (48%) groups than in White British (29%).

The last row in Table 1 shows the proportion of patients with Alzheimer's disease and mixed dementia who received cholinesterase inhibitor drugs. This did not differ by ethnic group χ^2^ = 1.11 (2 d.f.), *P* = 0.57.

As shown in Table 2, White British groups were more likely than Asian groups to receive a diagnosis of dementia: OR = 1.99 (95% CI 1.2–3.22, *P*=0.005) but compared with Black groups the difference was not significant (OR = 1.20, 95% CI 0.64–2.26, *P* = 0.56). Comparing Black and Asian groups, the former had a higher proportion diagnosed with dementia, but this was not statistically significant (odds ratio 1.65 (0.93–2.91), *P* = 0.08. Age and year of referral were significant predictors (with lower odds of confirmed dementia in more recent years).

**Table 2:** Random intercepts and slopes logistic regression model (patients within practices) predicting odds of having any dementia versus other diagnosis^a^

	Beta (95% CI)	Odds ratio (95%)	*P*	Interpretation of effect
Age	0.069 (0.046 to 0.091)	1.07 (1.04 to 1.10)	<0.001	7% increase in odds of dementia diagnosis for each extra year of age
Gender	0.084 (−0.33 to 0.55)	1.09 (0.71 to 1.65)	0.69	Non-significant 31% lower odds of dementia in females
Year of diagnosis	−0.13 (−0.26 to −0.06)	0.88 (0.77 to 0.994)	0.04	12% decrease in odds of dementia for each extra year
Ethnic group			0.012	Overall *F*-test significant (χ^2^ = 8.80), 2 d.f.
White British versus Asian groups (reference)	0.69 (0.20 to 1.17)	1.99 (1.23 to 3.22)	0.005	99% higher odds of dementia in White British. Significant after Bonferroni adjustment
White British versus Black groups (reference)	0.19 (−0.44 to 0.82)	1.20 (0.64 to 2.26)	0.56	Non-significant 20% higher odds of dementia in White British
Asian groups (reference) versus Black groups	0.50 (−0.07 to 1.07)	1.65 (0.93 to 2.91)	0.08	Non-significant 65% higher odds of dementia in Black groups

a. Patients missing data for the outcome or covariates or practice ID (12.6%) were excluded from the analysis. *n* = 568, 53 general practices. Analyses carried out in Stata 14 using MELOGIT.

On univariable analysis, compared with the White British population (5.7%), other or unstated dementia was more common in Asian (18.1%) and Black groups (29.2%), and the proportion of dementias diagnosed as Alzheimer's disease correspondingly lower. The proportion with vascular dementia was similar across groups but mixed dementia was lower in Black groups.

As shown in Table 3, of those with dementia, White British groups had a significantly higher adjusted odds of Alzheimer's dementia than Asian groups (OR = 1.76 (95% CI 1.11– 2.77), *P* = 0.015) but the overall ethnic group effect was borderline non-significant, so this result should be treated with caution. The other two pairwise ethnic differences did not reach statistical significance. In this model, earlier referrals (closer to 2012) were more likely to be classified as Alzheimer's disease than later ones. Year of referral was a significant predictor of a diagnosis of Alzheimer's disease, with lower odds in more recent years.

**Table 3:** Random intercepts and slopes logistic regression model (patients within practices) predicting odds of having Alzheimer's dementia vs other dementia^a^

	Beta (95% CI)	Odds ratio (95%)	*P*	Interpretation of effect
Age	−0.016 (−0.040 to 0.01)	0.98 (0.96 to 1.01)	0.16	Non-significant 2% decrease in odds of Alzheimer's dementia for each extra year of age
Gender	−0.29 (−0.69 to 0.12)	0.75 (0.50 to 1.13)	0.17	Non-significant 25% lower odds of Alzheimer's disease in females
Year of diagnosis	−0.15 (−0.26 to −0.03)	0.86 (0.77 to 0.97)	0.01	14% decrease in odds of Alzheimer's disease for each extra calendar year
Ethnic group			0.051	Overall *F*-test not significant (χ^2^ = 5.97). 2 d.f.
White British versus Asian groups (reference)	0.56 (0.11 to 1.02)	1.76 (1.11 to 2.77)	0.015	76% higher odds of Alzheimer's disease in White British
White British versus Black groups (reference)	0.23 (−0.34 to 0.80)	1.26 (0.71 to 2.23)	0.42	Non-significant 26% higher odds of Alzheimer's disease in White British
Asian groups (reference) versus Black groups	0.33 (−0.24 to 0.91)	1.39 (0.78 to 2.47)	0.25	Non-significant 39% higher odds of Alzheimer's disease in Black groups

a. Patients missing data for the outcome or covariates (<4%) were excluded from the analysis. *n* = 409, 49 general practices. Analyses carried out in Stata 14 using MELOGIT.

In order to model predictors of severity of diagnosis, moderate and severe categories were combined because of very low numbers of severe diagnoses in the Black group, which led to some empty cells in the regression matrix. Results are shown in Table 4.

**Table 4:** Random intercepts and slopes logistic regression model (patients within practices) predicting odds of having moderate/severe dementia versus mild dementia

	Beta (95% CI)	Odds ratio (95%)	*P*	Interpretation of effect
Age	0.013 (–0.012 to 0.038)	1.01 (0.98 to 1.04)	0.32	Non-significant 1% increase in odds of moderate or severe dementia for each extra year of age
Gender (reference female)	−0.512 (−0.94 to −0.082)	0.60 (0.39 to 0.93)	0.019	40% lower odds of moderate or severe dementia in females
Year of diagnosis	−0.19 (−0.32 to −0.07)	0.83 (0.72 to 0.93)	0.002	18% decrease in odds of moderate or severe dementia for each extra calendar year
Ethnic group			0.051	Overall test not significant (χ^2^ = 5.97). 2 d.f.
White British versus Asian groups (reference)	0.01 (−0.53 to 0.55)	1.01 (0.59 to 1.73)	0.97	Non-significant 1.01 times higher odds of moderate or severe dementia in White groups
White British versus Black groups (reference)	0.71 (0.09 to 1.32)	2.03 (1.10 to 3.72)	0.02	103% higher odds of moderate or severe dementia in White groups. Not significant after Bonferroni adjustment (*P* > 0.017)
Asian groups versus Black groups (reference)	0.70 (0.05 to 1.34)	2.01 (1.05 to 3.81)	0.03	101% higher odds of moderate or severe dementia in Asian groups. Not significant after Bonferroni adjustment (*P* > 0.017)

a. Patients missing data for the outcome or covariates (<4%) were excluded from the analysis. *n* = 409, 49 general practices. Analysis carried out in Stata 14 using MELOGIT.

Increasing age did not significantly increase the odds of being in a higher severity (moderate/severe) category compared with a mild category. Gender affected the odds of being in a higher severity category: women had 67% higher odds of being in a higher severity category. Ethnicity also appeared to significantly affect the odds of being in a higher severity category, although the overall effect of the ethnic group variable was non-significant (*P* = 0.051): *post hoc* tests showed that White British had 103% higher odds than Black groups, and Asian groups had 101% higher odds than Black groups, although with a Bonferroni correction these differences were not quite significant. There was no significant difference in severity at diagnosis between Asian and White British groups. With the passing of each extra year between 2010 and 2017, the odds of being in a higher severity category decreased by 17%. The effect was linear, the quadratic effect being non-significant when tested,

## Discussion

### Main findings

For referrals of White British people, 74.5% had dementia confirmed, similar to in Black groups (73.3%). In Asian groups, however, this was somewhat lower (61.5%) with higher proportions being diagnosed with mild cognitive impairment and depression. Compared with Asians, White British had twice the adjusted odds of being diagnosed with dementia. Although the number of Black patients was small, the proportion diagnosed with psychosis was highest in this group, with this difference reaching statistical significance.

Of those with dementia, unstated or other dementia was less common in White British (5.7%) than Asian (18.1%) and Black (29.2%) groups. A diagnosis of Alzheimer's disease was more common in White British (57.0%) than in Asian (43.8%), adjusted OR = 1.76 (95% CI 1.1–2.8). In total, 51% of Black patients were diagnosed with Alzheimer's disease, not significantly different from White British patients. It is reassuring that the use of Alzheimer's drugs for those who could benefit from them (i.e. with Alzheimer's disease or mixed dementia) did not differ by ethnicity.

After controlling for age, gender and year of diagnosis, we found no differences between Asian and White British groups in severity at presentation, but Black groups were less likely to have severe or moderate dementia compared with each of the other two groups, although after applying a Bonferroni correction this was not statistically significant. These findings are in contrast to two studies from London cited in the introduction, which found lower cognitive scores at presentation in Black and Asian groups^5^ and Black groups.^6^ This apparent discrepancy is likely to be because of differences within broad ethnic groups between Leicester and London.

These findings suggest GPs may respond to the difficulty of assessing cognition by referring more people from some BAME groups where there is uncertainty. The data on type of dementia suggests that specialists may also have more difficulty in distinguishing type of dementia in some BAME groups. This could explain why vascular and mixed dementia were not more often diagnosed in Black and Asian groups, despite higher prevalence of vascular risk factors in these populations.^17^

Our paper highlights the difficulties in differentiating the clinical heterogeneities in presentation at primary care, the difficulties in relying on standardised cognitive screening tools (including the widely used MMSE) in ethnic populations and the difficulties in delivering culturally appropriate interventions. Most services are agnostic to the cultural complexities of assessing and providing care and support to these groups. Arguably such issues may be found to a greater extent in other regions of the country where BAME groups may be less represented. Our study emphasises the need to raise awareness of such issues and possibly the need to provide ongoing training to address them even if this may seem to be only of limited scale and scope.

Further research is needed to further explore the reasons for the differences we found in rates of dementia in referrals from Asian and Black groups, including qualitative work examining GPs' decision to refer and the referral process from the perspective of patients and their families. Taken together this work will allow the development of guidelines to assist GPs in their assessment of cognition in these ethnic groups.

### Strengths and weaknesses

The major strength of the study is that we examined a large number of referrals to a memory clinic in an ethnically diverse city over several years. We were able to assess not only severity of dementia but also type of dementia and the prevalence of other diagnoses. However, the study has several limitations. First, MMSE scores were not recorded consistently in the NHS database used and so we had to rely on clinical review of the records, which could be considered less objective. It should be noted, however, that clinical assessment of severity may be more meaningful than cognitive scores,^18^ especially in comparisons between ethnic groups. Second, the reviewers could not be masked to ethnicity as they retrieved the clinical record using names. Third, we used broad ethnic groups to allow quantitative comparisons, which may have masked important differences within groups, for example in Asians between those from a Pakistani and Indian background. Fourth, we were unable to control for deprivation, as postcode for individuals was not reliably recorded and we felt that using GP postcode as a proxy would not be sufficiently sensitive.

More generally, a limitation of the whole study is that this is a single centre and so results cannot be generalised to places where the composition of the BAME population is different from in Leicester. This could explain why our findings differ from studies in London, where the composition of ethnic groups differs from Leicester. A number of results were non-significant, in spite of large OR (>1.65), suggesting the study was underpowered.

### Implications

The higher proportion of non-dementia diagnoses in Asian and Black groups suggest that GPs may respond to difficulties in assessment of cognition by having a lower threshold of referral. The higher proportion of unclassified dementia in these groups may be because specialists also find assessment more difficult. Unlike studies from other centres, we found no statistically significant evidence of greater severity at presentation in Asian and Black groups, although the large OR (OR = 2.03 White versus Black groups, and OR = 2.01 Asian versus Black groups) strongly suggest that Black groups have lower severity than each of the other two ethnic groups, with the non-significant results only occurring because the study was underpowered. An OR > 2 is highly likely to be clinically important.

## Data Availability

The data that support the findings of this study are available from the corresponding author upon reasonable request.
